# A Rare Subcutaneous Manifestation of Metastatic Renal Cell Carcinoma 

**DOI:** 10.1155/2016/6453975

**Published:** 2016-12-14

**Authors:** Darren Yak Leong Chan, Wei Jin Chua

**Affiliations:** Department of Urology, National University Hospital, Singapore

## Abstract

We report a rare case of advanced metastatic renal cell carcinoma which initially presented to the clinic with back and forehead lumps. Ultrasound imaging of the lumps and later of the abdomen picked up a right renal tumour which led to further computed tomography and bone scans. The bone scan confirmed that the forehead lump was a calvarial metastasis and such a presentation for metastatic RCC is very rare which bears a significantly poorer prognosis.

## 1. Introduction

Renal cell carcinoma (RCC) is often picked up incidentally on imaging [1] and may be advanced at time of diagnosis. The course is fairly indolent and patients are usually asymptomatic. RCC is thus characterised by various manifestations including unusual metastatic sites [2]. The classic presentation of haematuria, flank pain, and a palpable mass occurs in 20% of patients with RCC whilst up to 40% may present with a paraneoplastic syndrome [3]. Subcutaneous lump due to calvarial metastasis from RCC is very uncommon and in the case of our patient revealed an extensive metastatic burden.

## 2. Case Presentation

A 55-year-old man first presented with lumps on his mid-lower back and right forehead to his general practitioner who referred him to the General Surgery Department. On further history taking, it was noted to be associated with intermittent constipation, early satiety, and loss of weight of 4 kg over the duration of one month. There were no complains of gross haematuria or abdominal pain. He had no past medical history but had a 40-pack-year history of smoking. The lumps were approximately 4 cm in diameter, mobile, and painless with no surrounding erythema.

Gastroscopy and colonoscopy were performed, to evaluate the cause of the early satiety and constipation in a male aged above 50, which revealed polyps of tubular adenoma histology. Blood tests revealed normal renal function and electrolytes with elevated alanine transaminase and alkaline phosphatase. Ultrasound of the forehead lump was reported as a heterogeneous soft tissue lesion with skull vault destruction, highly vascular and separate from underlying brain parenchyma. The mid-lower back lump was reported as a solid vascular lesion. The radiologist decided to also perform a targeted ultrasound abdomen which located a right renal neoplasm with extension of the likely tumour thrombus into the right main renal vein and to the inferior vena cava. The patient was subsequently referred to the Urology Department which ordered further imaging to stage the tumour.

**Figure 1:**
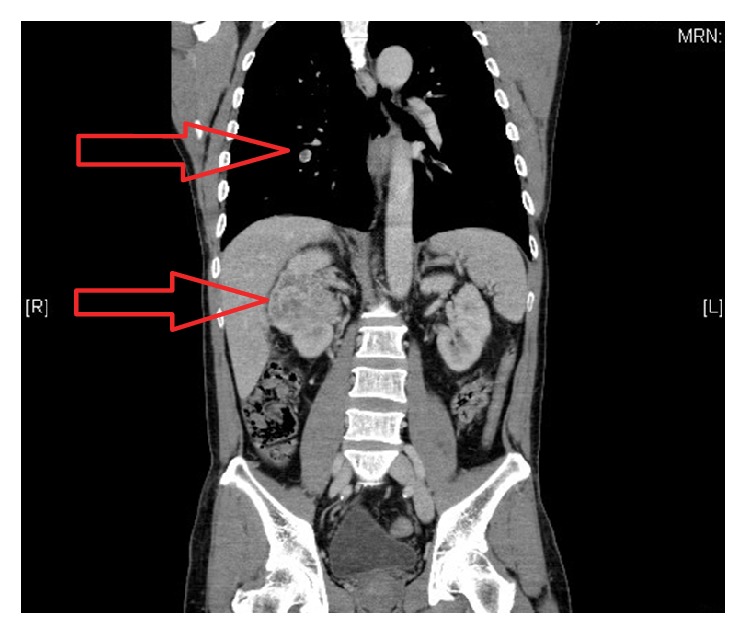
Right renal interpolar tumour with bilateral pulmonary metastases, worse on the right side.

**Figure 2:**
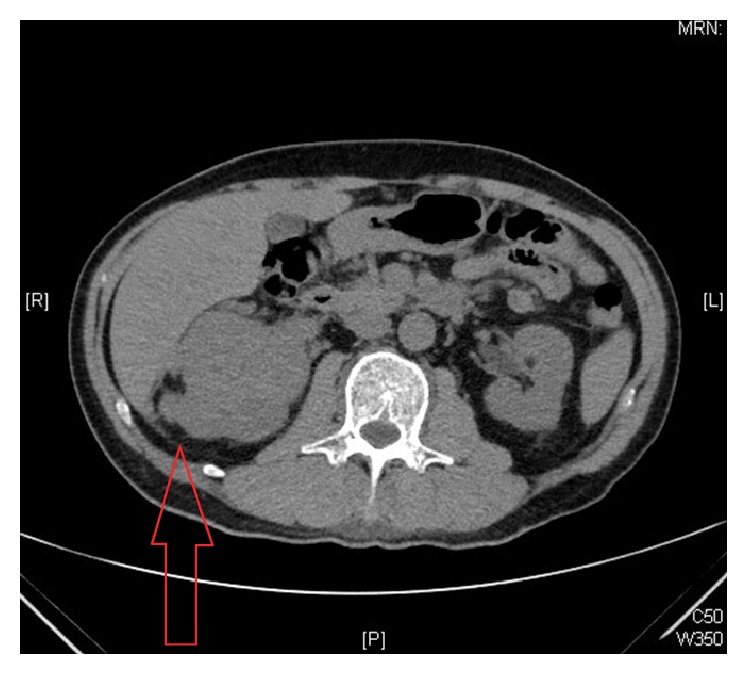
Focal invasion of right renal tumour into segment 6 of the liver.

**Figure 3:**
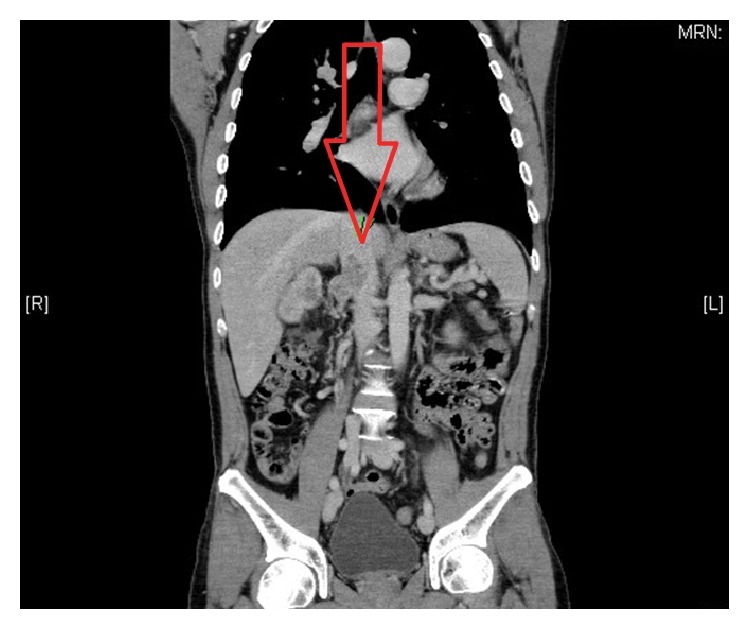
Tumour thrombus in the renal vein extending to the inferior vena cava up to the level as it enters the liver.

**Figure 4:**
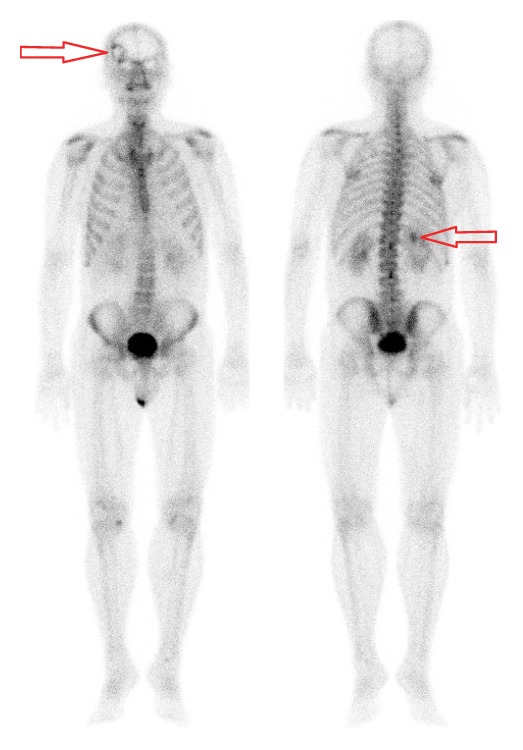
Bone scan showing right calvarial metastatic deposit and indistinct photopaenia at interpolar region of right kidney corresponding to primary tumour.

A computed tomography showed a 6.4 cm endophytic hypervascular right renal tumour (Figure 1) at the interpolar region with focal invasion into the liver (Figure 2) and seeding into the perinephric space and Gerota's fascia. It also confirmed the tumour thrombus in the renal vein extending into the inferior vena cava (Figure 3) and bilateral pulmonary arteries with pulmonary metastases (Figure 1). There was no lymphadenopathy noted. The back lump corresponded to the metastatic deposit which replaced the whole L2 spinous process without invading the spinal canal. On the bone scan (Figure 4), the forehead lump corresponded to the large photopaenic defect at the frontal region with increased osteoblastic activity suspicious of metastasis.

Based on the abovementioned imaging, the tumour was staged at T4N0M1, clinical stage IV. Consolidation cytoreductive nephrectomy was initially entertained with presurgical course of tyrosine-kinase inhibitors but at the multidisciplinary meeting it was decided that the patient was unlikely to benefit from cytoreductive nephrectomy due to the extensive metastatic burden with poor overall prognosis and the surgical risk was high in view of the bilateral pulmonary arterial thrombi. These options were still conveyed to the patient including the stage of his disease as well as the prognosis. Histological confirmation was also encouraged in the form of a fine-needle aspiration of the forehead or back lump. The patient refused to go ahead with any of the suggested procedures or any form of chemotherapy. He opted for Traditional Chinese Medicine and was subsequently referred for palliative services. The patient passed on 6 months later.

## 3. Discussion

More than 70% of renal cancers are picked up incidentally [1] and common sites of metastases include adrenals, intestines, lungs, and brain. Only five cases of calvarial mass have been reported as the first presentation of metastatic RCC [4] and rarely as skin manifestations, which bear a poorer prognosis [5]. Such presentations are often at advanced stages of disease and one should be highly suspicious of primary internal organ malignancy [6].

3–7% of patients with RCC have cutaneous metastases [7] and RCC itself corresponds to the primary tumour in 6–6.8% of all cutaneous metastases [6, 8, 9]. Cutaneous metastases may also be the initial presentation in 10–20% of cases [7]. Such metastases usually form multiple lesions [10] but isolated, solitary skin lesions as the primary presentation of the underlying RCC have been described in less than 20 cases to date in English literature [6, 11, 12]. Preferential involvement of the scalp with metastases from RCC has also been observed by others [13]. It is hypothesized that the predilection for the involvement of the face and scalp may be the result of valveless veins distributed in the head and neck region. Once the tumour cells disseminate, the presence of systemic shunts and tumour-associated growth factors also favours their deposition in this area. The normal pathway is usually through the lungs and hence patients with head and neck metastasis often have pulmonary metastases as well. Dissemination via the vertebral venous plexus explains the absence of pulmonary metastases in those with isolated head and neck metastasis [5, 14].

The cutaneous, head, and neck metastases usually represent a very poor prognosis with a reported survival period of less than 6 months [5, 14, 15]. The management of such advanced disease is controversial. Palliative nephrectomy can be offered in symptomatic patients with aggressive surgical resection of metastatic lesions. Radiotherapy alone or with surgery and systemic or intralesional interferon therapy have also been proposed as other options [5, 6, 16, 17].

Many large retrospective studies have reported the incidence of RCC with cutaneous metastasis and the different sites of cutaneous involvement and if there were other organs involved [18–22]. No details of outcomes were highlighted in them. Several single case studies highlighted the outcomes which were mostly poor but these patients had either multiple skin lesions or metastasis in other organs or were cases with recurrent RCC. These studies revealed a poor prognosis in patients with skin metastases at the time of diagnosis as it signified advanced disease [23–29]. Another study observed a patient with a clinical stage IV RCC who initially presented with a solitary lip cutaneous metastasis but passed on 4 months after receiving his first cycle of interferon treatment and postpalliative lip tumour resection [30].

However, there are 2 case reports in literature where patients with solitary skin lesions have been reported to be stable for more than 6 months after targeted chemotherapy was delivered [14, 31]. Another case was reported to have been disease-free two and a half years after a left radical nephrectomy, intralesional interferon, and targeted radiotherapy to the cutaneous metastasis at the chin and a subsequent lump resection. This case appeared to have a good outcome as this was the only solitary cutaneous metastases to the chin with no other organ involvement and he had received prompt treatment [6]. It was unfortunate that our case had extensive metastatic tumour burden at the time of diagnosis and that the patient was not keen for any treatment offered.

In conclusion, RCC can present with atypical sites of metastasis. Metastasis at the time of diagnosis of RCC frequently occurs, with the skin not infrequently involved. Solitary cutaneous metastases are uncommon but one should have a high index of suspicion for primary visceral malignancies upon detecting them. Prompt diagnosis and treatment may improve outcomes.
